# The effect of testosterone and a nutritional supplement on hospital admissions in under-nourished, older people

**DOI:** 10.1186/1471-2318-11-66

**Published:** 2011-10-24

**Authors:** Cynthia Piantadosi, Renuka Visvanathan, Vasi Naganathan, Peter Hunter, Ian D Cameron, Kylie Lange, Jonathan Karnon, Ian M Chapman

**Affiliations:** 1 The Health Observatory, University of Adelaide, Department of Medicine, Adelaide SA 5005, Australia; 2 University of Sydney, Concord Hospital, Concord NSW 2139, Australia; 3 Alfred Health, Caulfield Hospital 260, Caulfield Vic 3162, Australia; 4 Rehabilitation Studies Unit, University of Sydney, Ryde NSW 1680, Australia; 5 University of Adelaide, Department of Medicine, Adelaide SA 5005, Australia; 6 University of Adelaide, Department of Public Health, SA 5005, Australia

## Abstract

**Background:**

Weight loss and under-nutrition are relatively common in older people, and are associated with poor outcomes including increased rates of hospital admissions and death. In a pilot study of 49 undernourished older, community dwelling people we found that daily treatment for one year with a combination of testosterone tablets and a nutritional supplement produced a significant reduction in hospitalizations. We propose a larger, multicentre study to explore and hopefully confirm this exciting, potentially important finding (NHMRC project grant number 627178).

**Methods/Design:**

One year randomized control trial where subjects are allocated to either oral testosterone undecanoate and high calorie oral nutritional supplement or placebo medication and low calorie oral nutritional supplementation. 200 older community-dwelling, undernourished people [Mini Nutritional Assessment score <24 and either: a) low body weight (body mass index, in kg/m^2^: <22) or b) recent weight loss (>7.5% over 3 months)]. Hospital admissions, quality-adjusted life years, functional status, nutritional health, muscle strength, body composition and other variables will be assessed.

**Discussion:**

The pilot study showed that combined treatment with an oral testosterone and a supplement drink was well tolerated and safe, and reduced the number of people hospitalised and duration of hospital admissions in undernourished, community dwelling older people. This is an exciting finding, as it identifies a treatment which may be of substantial benefit to many older people in our community. We now propose to conduct a multi-centre study to test these findings in a substantially larger subject group, and to determine the cost effectiveness of this treatment.

**Trial registration:**

Australian Clinical Trial Registry: ACTRN 12610000356066

## Background

Ageing is associated with a physiological reduction of appetite and food intake [1,2]. Mean body weight decreases after about 60 years [3,4], largely due to a loss of muscle mass [5]. This age-associated loss of muscle mass and strength is called sarcopenia [6,7], which is present in up to 6-15% of older people [7]. Sarcopenia is associated with an increased risk of falls, disability and mortality [8-11]. The combination of muscle loss and reduced food intake predisposes older people to develop under-nutrition, which occurs in up to 10-15% of community-dwelling older people and many more in nursing homes [2,9]. Low body weight [10] and weight loss, particularly if involuntary, are major markers of harmful under-nutrition in older people [9,11].

In frail, malnourished, elderly people in nursing homes or hospital, daily oral nutritional (often high protein) supplementation has been shown to result in increased energy intake and body weight, reduced post-operative complications and length of hospital stay, and even reduced mortality [12,13]. The benefits of nutritional supplements have not been clearly established in frail, community-dwelling older people [14]. Circulating androgen concentrations decline in both men [15] and women [16] with increasing age, and there is increasing evidence that this contributes to the development of sarcopenia and the declining functional status that occurs with ageing [17,18]. Testosterone administration to older men has been shown to increase muscle mass and decrease fat mass [19-21]. In addition, it has been shown to increase muscle strength, albeit modestly and variably, more with high doses and in men with low testosterone levels [20,22,23]. In postmenopausal women testosterone treatment increases lean body mass, energy and libido [24,25] and possibly muscle strength [24]. We are unaware of evidence that testosterone improves clinically relevant outcomes, such as physical performance or cognitive function, in older women [24,26]. There have been a few reports of functional benefits of testosterone administration to older men; testosterone in high doses has been reported to improve standing ability after knee replacement surgery [27], improve timed walking[20] and increase the Function Independence Measure and grip strength [21]. Whilst androgen replacement therapy is advocated for men with low testosterone concentrations and symptoms of marked androgen deficiency, there is no consensus for its use in elderly men with less severe ageing-related declines in androgen concentrations, or in elderly women.

Hospitalisation is common in older people and imposes major burdens on the individual and community. Frail older people and those at nutritional risk are at increased risk of hospitalisation [28,29]. Our research group have previously reported that 43.2% of community dwelling recipients of domiciliary care services in Adelaide were under-nourished (38.4% of subjects at risk of malnutrition, 4.8% malnourished). 45% of these undernourished people were hospitalised in the subsequent year, a significantly higher rate than in well-nourished controls, with a 3-fold higher rate of hospitalization for more than one month [29]. As well as indicating the presence of an illness or illnesses sufficiently serious to require hospital admission, hospitalisation in older people is associated frequently with complications such as delirium, declining muscle strength and respiratory function, and is often followed by functional decline [30]. In older people there is a strong relationship between hospitalisation and the later development of disability, including disability severe enough to require moving to residential aged care [28,31]. Disability in older people is strongly associated with increased morbidity and mortality [32], is present in approximately 25% of those 74-85 years and 50% of those 85 years or older, and places substantial financial burdens on health care systems [33]. Hospital admissions is said to account for the majority of health-care costs in developed countries. In Australia and the USA people aged over 75 years spend 7-10 times as much time in hospital as adults under 45 years [34],[35]. This is likely to increase in coming years due to the ageing of the population and hospital bed days are predicted to double by 2050 [34].

It has been hypothesised that improving nutritional health through the administration of oral testosterone and nutritional supplementation to community dwelling older people over a period of 12 months may result in a reduction in hospitalisation and this will not only result in a reduction in health care costs but also an improvement in quality of life. Our research group have previously published exciting pilot data from a small study that provides further support for this hypothesis [36]. The Australian National Health and Medical Research Council has now funded this larger study to further investigate these findings.

Therefore, the aims of this randomized control trial is to determine the effects of treatment for one year with testosterone and high calorie nutritional supplementation compared to placebo testosterone and low calorie nutritional supplementation on the rate of hospital admissions and other endpoints in under-nourished, community-dwelling, older men and women.

## Methods/Design

The study has been approved by the Human and Research Ethics Committee of the Queen Elizabeth Hospital, Adelaide, South Australia. The study has been registered with the Australian Clinical Trial Registry: ACTRN 12610000356066.

### Recruitment and eligibility

Undernourished men and women, aged ≥ 65 years and living independently in the community, will be recruited in New South Wales, Victoria and South Australia (one site in each state) by several methods:

a) Domiciliary geriatric assessment services at the three campuses will approach new clients about their willingness to consider participation in this study and be approached by researchers.

b) Using similar approaches, investigator clinicians and other geriatrician/gerontology colleagues will refer consenting subjects from their rehabilitative, ambulatory or outreach services for further contact and review by the research officer. Any subjects who have only recently been in hospital will be enrolled into the study 3 months after the last hospitalization and once health status is stabilized.

c) Television, radio or newspaper advertisements.

Information about the research study will be posted to interested participants. Subjects will then be telephoned by the research officer and will only proceed if the subject consents to further discussions occurring over the telephone. Some preliminary screening questions will occur over the telephone to determine eligibility (i.e. recent weight loss and estimated weight and height). If subjects appear eligible and consent, the research officer will visit the subjects at home or the subjects will come to a clinic or a combination of these methods to determine subject eligibility for the study.

### Primary and Secondary Outcomes

The primary endpoints in this study are number of days in hospital, number of admissions and quality adjusted life years (QALYs). Secondary endpoints are measures of function, nutrition, strength, body composition, inflammation and health service utilization.

### Inclusion and Exclusion Criteria

In this study, the subject is said to be at-risk of under-nutrition if they fulfill the following:

a) A Mini Nutritional Assessment (MNA) score between 17 and 23.5 [37]; and

b) A body mass index (in kg/m^2^) of <22 or a self-reported weight loss of ≥7.5% in the 3 months before enrolling in the study.

The following exclusion criteria are applied:

a) The inability to comply with the protocol;

b) Folstein's Mini Mental State Examination score ≤23 [38];

c) Elevated hematocrit (>50%); history of prostate cancer, prostate-specific antigen (PSA) concentrations greater than the age-related normal range, or an irregular prostate on examination;

d) A history of breast cancer in men and women;

e) Preexisting androgenic signs or symptoms of concern (deep voice, hirsutism, acne, or androgenic hair loss) in women;

f) Significant depressive symptoms using the Geriatric Depression Scale (short form) score ≥11 [39];

g) Cardiac failure corresponding to New York Heart Association class III and above;

h) Myocardial infarction or stroke within the past 12 months, unstable angina, coronary artery procedure (stent, angioplasty or coronary artery bypass grafting) within the past 12 months, unstable arrhythmia (does not include controlled atrial fibrillation);

i) Uncontrolled hypertension; systolic blood pressure >170 mmHg and/or diastolic blood pressure >100 mmHg

j) Abnormal liver function tests (alanine aminotransferase, *γ*-glutamyltransferase, bilirubin, or alkaline phosphatase >2 times the upper limit of normal;

k) Estimated creatinine clearance < 30 ml/min (by the equation of Baracskay and Jarjoura for ambulatory elderly subjects [Cr clearance = 4.4/serum creatinine (mmol/L) + (88-age)] AND/OR serum creatinine concentration > 0.2 mmol/l. [40]

l) Any disease that, in the opinion of the investigator, is likely to lead to death within 1 year;

m) Testosterone or other androgen therapy within 4 months of starting the study; and

n) Women on oestrogen or hormone replacement therapy that have not been on a stable dose for the last 3 months.

**Figure 1:**
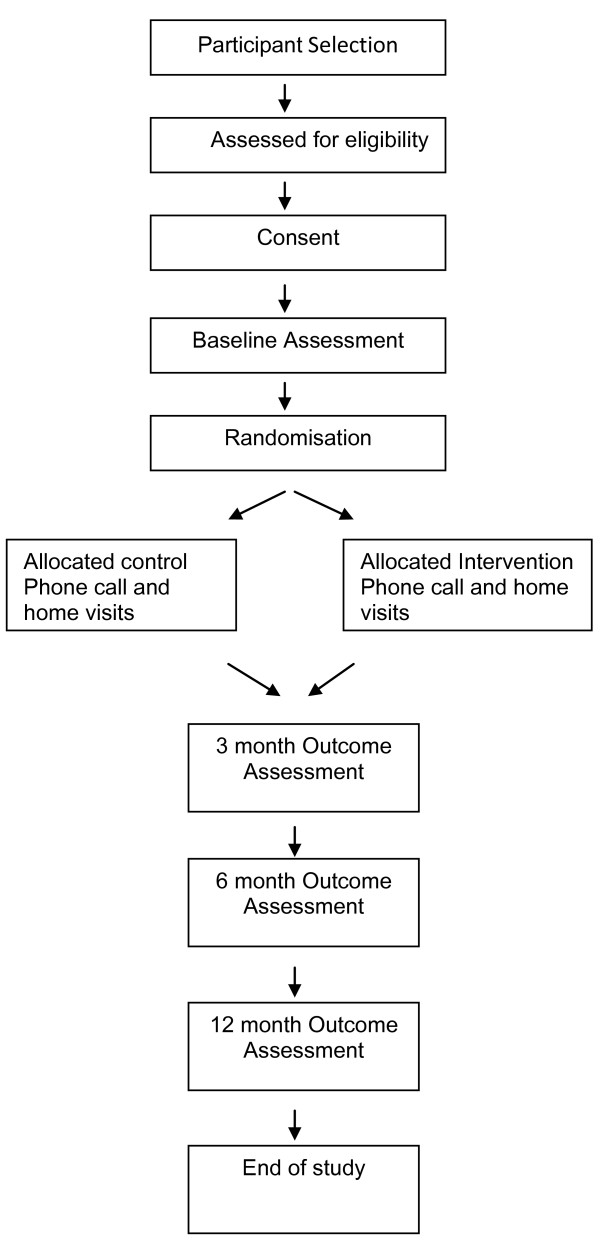
**Study design and assessment points**.

All subjects will give written informed consent prior to commencement in the study. Subjects are able to withdraw from the study at any time. Appropriate security and de-identification measures will be used to ensure subject confidentiality. An overview of the recruitment method, randomization and following-up time points is shown in Figure 1.

### Randomization

Subjects will be randomized to one of two study groups (n = ≈100/group) by a stratification system to ensure that an equal number of men and women will be allocated to each treatment. There will be stratification by study site to allow for possible differences in subjects recruited between sites, in blocks of 10 within each sex at each site. To maintain blinding a master randomisation list will be developed and maintained by the Research Pharmacy Department at the Royal Adelaide Hospital in Adelaide. Separate lists will be developed for each study site - the investigators will be blinded to these treatment lists.

### Interventions

All subjects will be advised that they have been assessed as at-risk of being under-nutrition, and will be given dietary advice about increasing their energy intake, based on their responses to the MNA assessment as in the previous study. In addition, the general practitioner of all subjects will be notified in writing of this assessment and of the subject's participation in the study. This will be designated 'standard care'.

The two study groups will be:

a) **Control**: "standard care" + placebo testosterone + placebo- low calorie supplement drink (< 200 kJ in 180 ml/day).

b) **Combined treatment**: "standard care" + oral testosterone undecanoate (Andriol Testocaps 40 mg orally once a day for women, 80 mg orally twice a day for men) + high calorie nutritional supplement (1084 kJ in 180 ml/day, approx 11 g protein, approx 10 g fat, approx 40 g carbohydrate).

Testosterone undecanoate capsules are purchased and a packaging company places these capsules in a larger capsule. These larger capsules minus the testosterone act as the placebo. Dissolution testing was conducted to ensure that absorption of the active testosterone medication was not significantly altered. Subjects are advised to take their testosterone/placebo tablets with meals (with breakfast and evening dinner for men and with breakfast for women). The high calorie nutritional supplement used in this study is a 180 ml drink that is taken twice a day. Two flavours are provided, Vanilla 1054 kJ/180 ml and Chocolate 1208 kj/180 ml. Subjects on the active treatment would receive approximately 2108-2416 kJ(360 ml)/day. The placebo consists of a low KJ (<200 KJ) drink made up of artificial sweetener, flavour and xantham gum to match the texture. The vanilla flavoured drink delivers 70.97 kJ/180 mls and the chocolate flavoured drink delivers 95.48 kJ/180 mls. Subjects on the placebo treatment would receive approximately 142-191 kJ (360 ml)/day.

At baseline the following assessments are conducted.

a. Nutritional assessment- completion of the Mini Nutritional Assessment (MNA) [37] and determination of body mass index (weight/height^2^) and the presence of weight loss. The Simplified Nutrition Appetite Questionnaire (SNAQ) [41] and a 24 hour recall food diary will also be administered;

b. Documentation of medical history and medications including the completion of the Charlson co-morbiditiy index [42];

c. Completion of the following validated questionnaires and assessments: Folstein's Mini Mental State Examination (MMSE) [38], Montreal Cognitive Assessment (MOCA) (http://www.mocatest.org)[43], 15-item geriatric depression scale (15-GDS) [39], Independent Activities of Daily Living scale (IADL) [44], Barthel's Index [45], the Cardiovascular Health Study Frailty Phenotype Score [46], a retrospective falls diary for the previous 12 months, a 24 hour food recall diary, the 36-Item Short Form-36 Health Survey (SF-36) [47], for men International Prostate Symptom Score [48], for women voice handicap questionnaire [49], and a record of hospital admissions over the previous year.

d. Completion of strength examination: 3 metre walk time, dominant hand grip strength (Stoelting Hand Grip Dynanometer; Stoelting Co, Wood Dale, IL), timed chair rise time (time taken to perform 5 chair rise and number performed in 30 seconds);

e. Digital rectal prostate examination (DRE) in men;

f. Dipstick urinalysis;

g. Body composition analysis by Bioelectrical Impedance (BIA: Quantum II by RJL Systems [http://www.rjlsystems.com/products]);

h. Biochemical analysis (fasting blood for glucose, lipids, hematocrit [HCT], PSA [men only], electrolytes, liver function tests, high sensitivity C-Reactive Protein (hsCRP), serum lipids, albumin, testosterone, sex hormone binding globulin [SHBG], FSH, LH, oestradiol); and

i. storage sample for later cytokine measurement (IL-1, IL-6, TNF-α).

Further assessments at home or clinic occur at 3-months, 6 months and 12 months as described in Table 1. The protocol will be continued during, and after, any hospitalization if this did not result in adverse effects or interfere with any medical treatment. Subjects will be visited at home at 3, 6, and 12 months for measurements of weight, anthropometry, and grip strength and at 1, 2, 3, 4, 5, 6, 8, 10, and 12 months for assessment of dietary and medication compliance (tablet and empty packet count) and documentation of any change in medical conditions, medications, living arrangements, or hospitalizations. Details of hospital admissions are obtained from the subject and, with their written permission, from their general practitioner. Where possible, hospital records, including copies of discharge letters, are obtained. Compliance is reinforced and assessed by phone calls every 2 weeks between visits. The occurrence of any adverse events is actively sought during these calls and visits.

**Table 1:** Proposed visits and measurements during the study

	Screening	Baseline	3 m	6 m	12 m
Screening Document	X				
Consent		X			
Baseline-demographics, illness, medications, Charlson co-morbidity index		X			
Chair rise test		X		X	X
Weight		X	X	X	X
IPSS (men only)		X	X	X	X
MNA, SNAQ		X		X	X
MMSE, MOCA, 15-GDS, IADL, Barthel Index		X		X	X
Voice Handicap Index (women only)		X	X	X	X
Frailty, Grip Strength, physical activity (kcal/wk)		X	Grip only	X	X
SF-36		X		X	X
Body composition-resistance, reactance, fat mass, lean mass, TBW		X			X
Urinalysis and per rectal exam for men	X				
24 hour food diary (%fat, % CHO, %Protein, kcal)		X		X	X
FBE (hematocrit)		X	X	X	X
PSA (men only)		X	X	X	X
Total Chol, Trig, HDL, LDL		X		X	X
Biochem		X		X	X
hsCRP		X		X	X
Testosterone, SHBG		X		X	X
LH, FSH, Oestradiol		X			X
Cytokines		X		X	X
Flag any abnormal blood test results to CI		X	X	X	X
Send results to GP		X	X	X	X

### Alterations to testosterone dosage

The study drug dose will be halved to one tablet twice a day in men, and one tablet alternate days in women if either of the following occur (confirmed by repeat test): hematocrit > 54%; PSA more than 10% above the upper end of the age-related normal range. The abnormal test will be repeated in 6 weeks. If the value normalizes the subject will continue the halved dose. If the result remains elevated the study medication will be ceased permanently (the abnormal value will be re-measured 6 weeks later and then according to the study schedule) and the subject will continue only with the nutritional supplement (or control). Men with persistently elevated PSA values will be referred for further urological assessment. Women who develop androgenic side effects have the option of stopping the drug or halving the dose. If the dose is halved the full dose will not be re-instituted later and the subject has the option of stopping the study drug at any later time.

### Data and Safety Monitoring

An independent data and safety monitoring committee, consisting of a nutritional expert/scientist, geriatrician and another specialist physician with experience on the drugs and therapeutics committee who are independent of the study investigations and unaware of the treatment assignments will be established and co-ordinated from the Queen Elizabeth Hospital. This committee will meet at regular intervals to monitor for adverse events that warrant protocol modification or termination. For instance, emergence of an unacceptable adverse effect profile with treatment which is different to that experienced in the pilot study. Because of the size of the study and the nature of the treatment and endpoints we have not included an option for premature study termination due to high efficacy.

### Statistical and Cost-Effectiveness Analysis

All analyses will be performed according to the intention-to-treat principle controlling for the effects of recruitment site. The distribution of the number of hospitalizations in each group will be compared using Fisher's exact test. Times to admission will be analyzed using Cox proportional hazards regression with account taken of within-subject correlation for subjects with multiple admissions. The numbers of days spent in hospital will be compared using Mann-Whitney tests. Other data will be analysed with a linear mixed model using residual maximum likelihood (REML) and including all available time points. A per-protocol analysis will also be undertaken. A cost-effectiveness analysis will be undertaken from the perspective of the Australian public health care system and include costs incurred by both the federal and state governments in relation to health care. The primary outcome measure for the cost-effectiveness analysis will be the quality adjusted life years (QALY) derived from SF_36 via the SF-6D algorithm [50]. Patients will be asked for consent to access their Pharmaceutical Benefits Scheme (PBS) and Medicare Benefits Schedule (MBS) records. Consent will also be requested to access patient information from the Integrated South Australian Activity Collection (ISAAC), which contains information on all hospital separations in SA. Consent will also be requested to access patient information from relevant State agencies which will contain information on all hospital separations in each State, and the related information on the costs associated with each hospital separation. Data obtained will include patient admission, and inpatient stay characteristics, as well as providing ICD-10 categorised data on principle and additional diagnoses and procedures. These three data sources will provide sufficient data to inform a detailed analysis of the downstream cost consequences for the different treatment groups.

Subjects will still be allowed to participate in the study without providing consent to access these data. These three data sources will provide sufficient data to inform a detailed analysis of the downstream cost consequences for the different treatment groups.

Mean costs and effectiveness between the intervention and control groups will be compared and incremental cost effectiveness ratios presented (ICERs) described. A range of one-way and multi-way sensitivity analyses will be undertaken to test the effect of uncertainties around the true values of key resource use and effectiveness parameters. Probabilistic sensitivity analysis will inform confidence intervals around the ICER and cost-effectiveness acceptability curves.

### Power and Sample Size

The sample size is based on power calculations performed on the results of the pilot study [36]. To have a power of 90% to detect a significant (at P = 0.05 [2-sided]) difference in the number of days of hospitalisation between the treatment groups 28 subjects per group are required, and to have a power of 90% to detect a significant (at P = 0.05 [2-sided]) difference in the number of subjects with non-elective admissions 30 subjects per group are required. The study is therefore substantially overpowered to address the primary endpoint of hospitalisation. An examination of QALYs in the pilot study indicates that the no-treatment group lost QALYs, while the combined treatment group gained QALYS. 85 subjects per treatment arm would be needed to have a power of 90% to show a significant (P = 0.05 [2-sided]) QALY improvement of 0.05 with the combined treatment. Assuming a net cost of providing the treatment of AUS$2500 per year when used in the community setting, a QALY difference of 0.05 would result in an incremental cost per QALY gained of AUS$50,000, the commonly implied threshold for cost effectiveness (Medicines Australia Inc, A Prescription for the Health of Australia 2002). Allowing for a margin of safety, we therefore plan to study 200 subjects.

## Discussion

There is limited high level evidence that any specific interventions are effective strategies for community dwelling older people identified as under-nourished who are at-risk of morbidity and mortality [29,51]. Under-nourished older people are likely to have sarcopenia and this provides the basis for the use of an anabolic agent such as oral testosterone. To date, the evidence for functional benefits of testosterone treatment in older people, other than increased strength, is limited. This research group has previously reported that 12 month combined treatment with oral testosterone and nutritional supplement reduced the risk of hospitalization compared to no treatment in a group of under-nourished community dwelling older people. Previous studies have reported that nutritional supplements have modest benefits in older people, particularly in those who are more undernourished, who are in hospitals or other institutions, or who receive larger dose of supplements for longer time periods [14,52]. This larger study will provide researchers with further information allowing determination of the effectiveness of this proposed intervention strategy in this group of population (frail, older, community dwelling and under-nourished).

## Competing interests

Professor Chapman and Associate Professor Visvanathan have received funding previously from Organon Pty. Ltd. for the pilot study. R.Visvanathan is a member of the Nestle Australia Healthcare Nutrition Malnutrition in the Elderly Board member and has received funding from Nestle as part of the Mini Nutritional Assessment initiative.

## Authors' contributions

The authors' responsibilities were as follows--IMC and RV: conception and design of study, preparation of ethics protocol and editing of protocol article, CP, drafting and revising of the protocol article; VN, PH, IC, KL, JK: drafting and revising of the article, and all authors final approval.

## Pre-publication history

The pre-publication history for this paper can be accessed here:

http://www.biomedcentral.com/1471-2318/11/66/prepub
